# Hydrogen Generation by Hydrolysis of MgH_2_-LiH Composite

**DOI:** 10.3390/ma15041593

**Published:** 2022-02-21

**Authors:** Xiaojuan Wu, Huaqing Xue, Yong Peng, Jifeng Deng, Zewei Xie, Jie Zheng, Xingguo Li, Shuan Li

**Affiliations:** 1 Beijing National Laboratory for Molecular Sciences, College of Chemistry and Molecular Engineering, Peking University, Beijing 100871, China; xiaojuanwu@pku.edu.cn (X.W.); jfdeng@pku.edu.cn (J.D.); zwxie@pku.edu.cn (Z.X.); zhengjie@pku.edu.cn (J.Z.); xgli@pku.edu.cn (X.L.); 2 State Key Laboratory of Rare Earth Materials Chemistry and Applications, College of Chemistry and Molecular Engineering, Peking University, Beijing 100871, China; 3 Research Center of New Energy, Research Institute of Petroleum Exploration & Development, Beijing 100083, China; hqxue@petrochina.con.cn (H.X.); pengyong13@petrochina.com.cn (Y.P.)

**Keywords:** MgH_2_-LiH composite, ball milling, hydrogen generation, bath temperatures, MgCl_2_ aqueous solution

## Abstract

As a most promising material for hydrogen generation by hydrolysis, magnesium hydride (MgH_2_) is also trapped by its yielded byproduct Mg(OH)_2_ whose dense passivated layers prevent the further contact of intimal MgH_2_ with water. In this work, LiH, as a destroyer, has been added to promote the hydrogen properties of MgH_2_. The results demonstrate that even 3 wt % LiH was added into MgH_2_-G, the hydrogen generation yield can increase about 72% compared to the hydrogen generation yield of MgH_2_-G. The possible mechanism is that Mg^2+^ from the hydrolysis of MgH_2_ preferentially bound with OH^−^ ions from the hydrolysis of LiH to form Mg(OH)_2_ precipitation, which is dispersed in water rather than coated on the surface of MgH_2_. Moreover, adding MgCl_2_ into hydrolysis solution, using ball milling technology, and increasing the hydrolysis temperature can make the hydrolysis rate higher and reaction process more complete. It is noted that a too high weight ratio of LiH with too high of a hydrolysis temperature will make the reaction too violent to be safe in the experiment. We determinate the best experimental condition is that the LiH ratio added into MgH_2_ is 3 wt %, the hydrolysis temperature is 60 °C, and the concentration of MgCl_2_ hydrating solution is 1 M. MgH_2_-LiH composite hydrogen generation technology can meet the needs of various types of hydrogen supply and has broad application prospects.

## 1. Introduction

Currently, the contradiction between the decreasing of global fossil energy and the infinite demand for energy is the main factor that restricts the sustainable development of society [1,2]. Hydrogen energy is considered to be the most potential energy carrier for replacing traditional fossil fuels in the future because of its advantages such as abundant reserves, high energy density (142 MJ/kg), environmental protection, and renewability [3,4,5,6]. Hydrogen fuel cell is the most attractive new energy, and its “fuel” is hydrogen, so efficient and safe hydrogen generation technology is particularly important [7]. Hydrogen generation by hydrolysis is a kind of on-site hydrogen generation method, which can be easily applied to various mobile devices [8,9,10].

At present, scientists have paid their attention into the hydrogen generation by the hydrolysis of metal or metal hydrides and chemical hydrides, such as MgH_2_, NaBH_4_, CaH_2_, and LiH, and their theoretical hydrogen yield of are 15.32 wt %, 21.32 wt %, 9.58 wt %, and 25.36 wt % (without H_2_O), respectively. NaBH_4_ is considered as a promising hydrogen storage material; however, NaBH_4_ needs noble catalysts to improve the kinetic performance of hydrolysis, and high cost of recovery of byproducts [11,12,13], while CaH_2_ and LiH all react violently with water, making it difficult to control the course of experiments [14,15].

By comparison, MgH_2_ has not only a high theoretical hydrogen generation but also environmentally friendly hydrolysis byproduct Mg(OH)_2_, what is more, the hydrolysis of Mg-based materials is considered as a clean hydrogen generation technique and Mg element is abundant on Earth [16]. MgH_2_ has a lower cost than other hydrolyzed materials, which is regarded the most promising hydrolysis of material. The reaction equations are as follows:

(1) $$\mathrm{Mg}+2H_{2}O\to \mathrm{Mg}{\left(\mathrm{OH}\right)}_{2}+H_{2}$$

(2) $${\mathrm{MgH}}_{2}+2H_{2}O\to \mathrm{Mg}{\left(\mathrm{OH}\right)}_{2}+2H_{2}$$

However, the dense passivation layer of Mg(OH)_2_ will prevent the further contact of water with intimal MgH_2_, which results in sluggish hydrolysis kinetics [17,18]. In the process of hydrolysis, in order to remove the passivation layer and promote the hydrolysis reaction of MgH_2_, researchers have made great efforts, such as the introduction of various cations/anions in the solution [19,20,21], the addition of additives [21,22,23], alloying [16,24,25,26,27], reduce Mg particle size [28], and so on. These methods play an important role in improving the hydrolysis kinetics and hydrogen generation capacity to a certain extent.

In this paper, to improve the hydrolysis properties of MgH_2_, we choose the LiH with high hydrogen storage capacity and light weight to add into MgH_2_. The effects of milling technology, solution, water solution temperature, and the amount of adding LiH on the hydrolysis performance of MgH_2_ were studied.

## 2. Experimental Details

### 2.1. Sample Preparation

The Mg powder was prepared by the hydrogen plasma metal reaction (HPMR), the detailed description is given in the published article [29]. Then, the Mg powder was placed in a steel reactor which vacuum to 10^−3^ Pa, and heated to 400 °C for 5 h, followed by hydrogenation under 4 MPa hydrogen atmosphere at 673 K for 10 h. Finally, the reactor was vacuumed, and the sample was taken out to obtain the MgH_2_. The MgH_2_ and LiH powders were mixed in different mass ratios by obtained by planetary ball milling (BM) and by grinding (G) in agate mortar, respectively, which operated in the argon atmosphere glove box. The LiH was purchased from Alfa Aesar (Lancashire, UK), there is no further purification.

The phase composition of the samples was determined by a powder X-ray diffraction (XRD) method. The XRD patterns were obtained on an X-Pert3 powder diffractometer (PANalytical, Almelo, The Netherlands) in the 2θ range from 5° to 80° using the CuKα radiation. The morphologies of the samples were observed using a JSM-IT300 (JEOL, Tokyo, Japan) scanning electron microscopy (SEM).

### 2.2. Hydrolysis Experiment

The hydrolysis reactions were tested on a self-assembled system in room temperature. The 50 mg of sample was added into the flask with three opening in the glove box and take out the glove box and quickly connect it to the test system. Then 50 mL of 1 M MgCl_2_ aqueous solution was injected into the conical flask with a syringe. After opening the peristaltic pump, MgCl_2_ aqueous solution dropped in the three-mouth flask, and the hydrogen was collected by draining water gathering of gas law and recorded the volume of discharged water.

## 3. Results and Discussion

**Figure 1:**
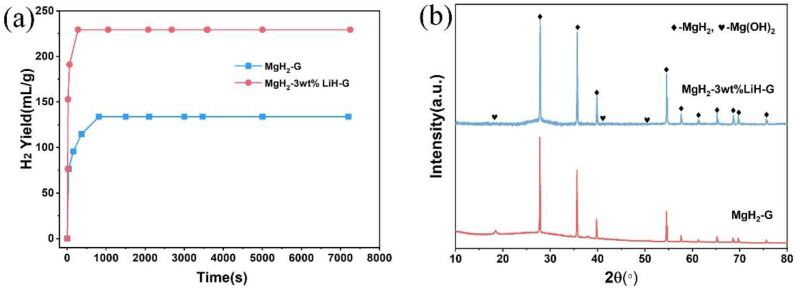
(**a**) The hydrogen generation curves and (**b**) XRD patterns of the hydrolysis byproduct of MgH_2_-G and MgH_2_-3 wt % LiH-G.

Figure 1a shows the hydrogen generation curves of MgH_2_-G and MgH_2_-3 wt % LiH-G in deionized water. As shown in Figure 1a, the hydrogen yield of the MgH_2_-G and MgH_2_-3 wt % LiH-G are about 130 mL/g, and 230 mL/g, respectively. Although the hydrogen generation yield of MgH_2_-3 wt % LiH-G and MgH_2_-G are only about 10% of their theoretical value, respectively, the hydrogen generation yield of MgH_2_-3 wt % LiH-G increase about 72% compared to the hydrogen generation yield of MgH_2_-G. At the same time, from Figure 1b, the hydrolysis byproducts mainly contain a major phase unreacted MgH_2_ and a secondary phase Mg(OH)_2_. The XRD results indicate that the Mg(OH)_2_ layer formed on the surface samples hindered the hydrolysis reaction. However, it also shows that the LiH addition increased the hydrogen yield properties.

**Figure 2:**
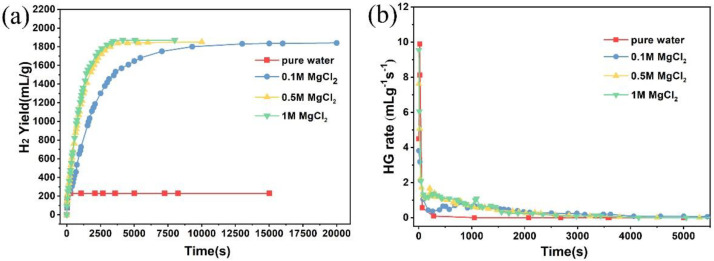
(**a**)The hydrogen generation curves and (**b**)hydrogen generation rate and hydrolysis by-product XRD patterns of MgH_2_-3 wt %LiH-G in 0.1 M, 0.5 M and 1 M MgCl_2_ solutions.

**Figure 3:**
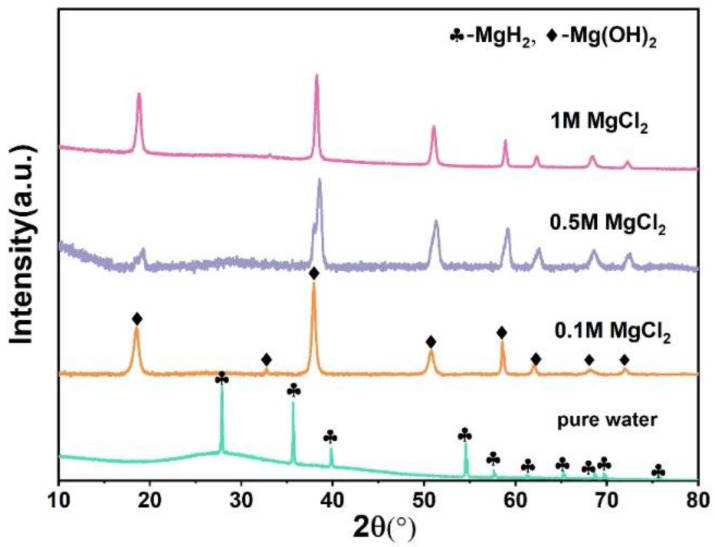
XRD patterns of the hydrolysis by-products of MgH_2_-3wt %LiH-G in the different concentrations of MgCl_2_ aqueous solution.

In order to improve the hydrogen yield, adding a small amount of MgCl_2_ is a common way to promote the hydrolysis of MgH_2_. Figure 2a,b are the hydrogen yield and the hydrogen generation rate of the MgH_2_-3 wt % LiH-G in the different concentrations (0, 0.1, 0.5, 1) of MgCl_2_ aqueous solution, respectively. As shown in Figure 2a, the hydrogen yield curves in 0.1 M, 0.5 M, and 1 M MgCl_2_ solutions are compared. The hydrogen production rate diagram from Figure 2b is converted from the data in Figure 2a. The results indicate that MgCl_2_ aqueous solution is beneficial for improvement of the MgH_2_-3 wt % LiH hydrolysis; their hydrolysis reaction is relatively complete, almost 100%. In addition, in the 1M MgCl_2_ aqueous solution, the sample has the fastest hydrolysis rate. The result is consistent with XRD results from Figure 3. The hydrolysis byproduct mainly contains Mg(OH)_2_; unreacted MgH_2_ is not found.

**Figure 4:**
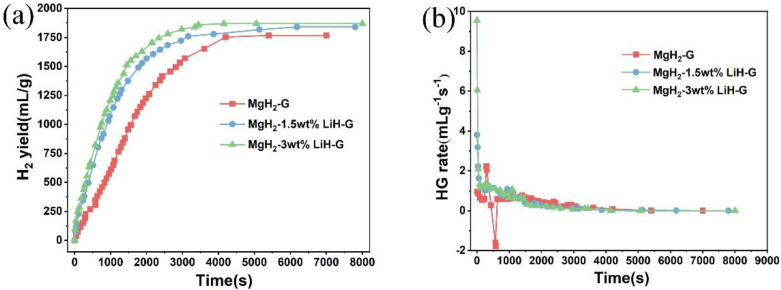
(**a**) Hydrogen generation curves and (**b**) hydrogen generation rate of MgH_2_-G, MgH_2_-1.5 wt % LiH-G, and MgH_2_-3 wt % LiH-G.

In order to obtain the effect of LiH on MgH_2_-LiH system, we tested the hydrogen generation yield and hydrogen generation rate of MgH_2_ with the different amounts of LiH, as shown in Figure 4a,b, respectively. From Figure 4a, the MgH_2_-G, MgH_2_-1.5 wt % LiH-G, and MgH_2_-3 wt % LiH-G generated 1753 mL/g, 1840 mL/g, and 1870 mL/g hydrogen, respectively. Obviously, the addition of LiH improves the hydrogen yield. Furthermore, we can see that the hydrogen generation rate is also improved in Figure 4b. The results indicate the addition of LiH not only enhance hydrogen generation yield but also increase hydrogen generation rate. The mechanism of the reaction will be described later in this paper.

**Figure 5:**
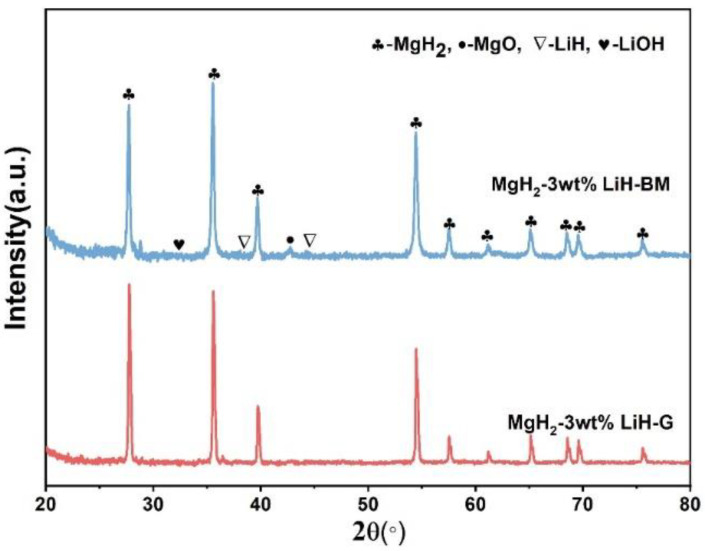
XRD patterns of the hydrolysis byproduct of MgH_2_-3 wt % LiH-BM and MgH_2_-3 wt % LiH-G.

Figure 5 shows the XRD pattern of MgH_2_-3 wt % LiH-BM and MgH_2_-3 wt % LiH-G. We compared the hydrolytic properties of MgH_2_-3 wt % LiH-BM and MgH_2_-3 wt % LiH-G. The two samples were composed of MgH_2_ and LiH, in addition, small peaks of MgO and LiOH could also be observed. This MgO is introduced during preparation and LiOH is introduced XRD characterization process.

**Figure 6:**
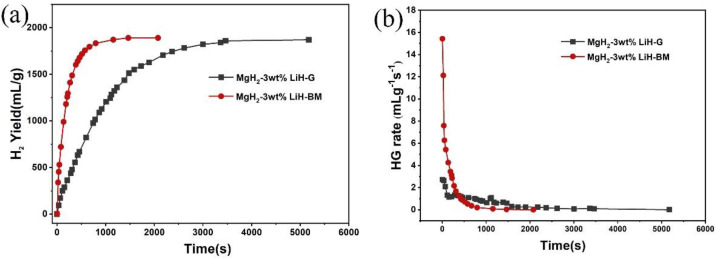
(**a**) Hydrogen generation curves and (**b**) hydrogen generation rate of MgH_2_-3 wt % LiH-BM and MgH_2_-3 wt % LiH-G.

**Figure 7:**
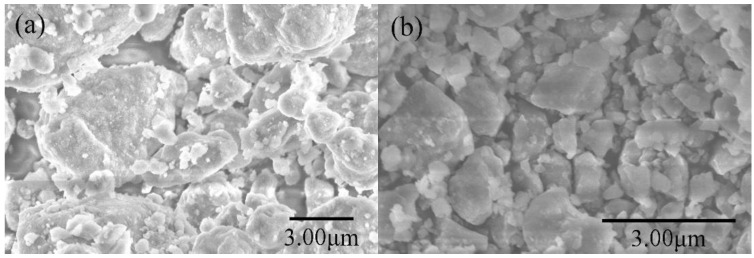
(**a**) SEM of MgH_2_-3 wt % LiH-G and (**b**) MgH_2_-3 wt % LiH-BM.

Figure 6a,b show the hydrogen generation curves and hydrogen generation rate of MgH_2_-3 wt % LiH-BM and MgH_2_-3 wt % LiH-G, respectively. It can be seen that the MgH_2_-3 wt % LiH-BM can generate about 1830 mL/g in 800 s, continue to generate hydrogen until 1890 mL/g in 1500 s, which can close to the maximum hydrogen yield for the studied samples. The MgH_2_-3 wt % LiH-G can generate 1820 mL/g in 3000 s. Finally, the maximum value is reached more than 3500 s. Obviously, the MgH_2_-3 wt % LiH-BM exhibits a higher hydrogen generation rate than the MgH_2_-3 wt % LiH-G. From the above experimental results, the ball milling in a short time plays an important role in increasing the rate of hydrogen generation. The results should be closely related to the particle size of the sample. In order to provide more direct experimental evidence, the SEM images of the two samples are given in Figure 7. From the SEM images of the two samples, we can see that the MgH_2_-3 wt % LiH-BM are obviously smaller than the MgH_2_-3 wt % LiH-G, proving the above hydrogen generation properties analysis results of two samples.

**Figure 8:**
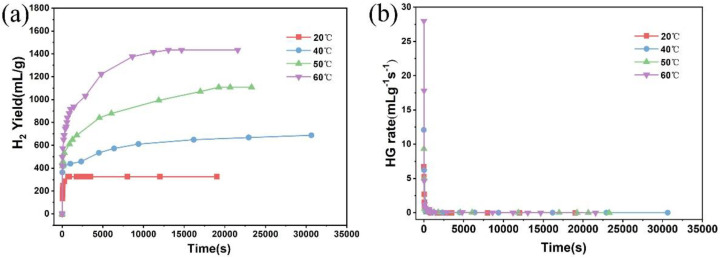
(**a**) Hydrogen generation curves and (**b**) hydrogen generation rate of the MgH_2_-3 wt % LiH-BM in water at different bath temperatures.

**Figure 9:**
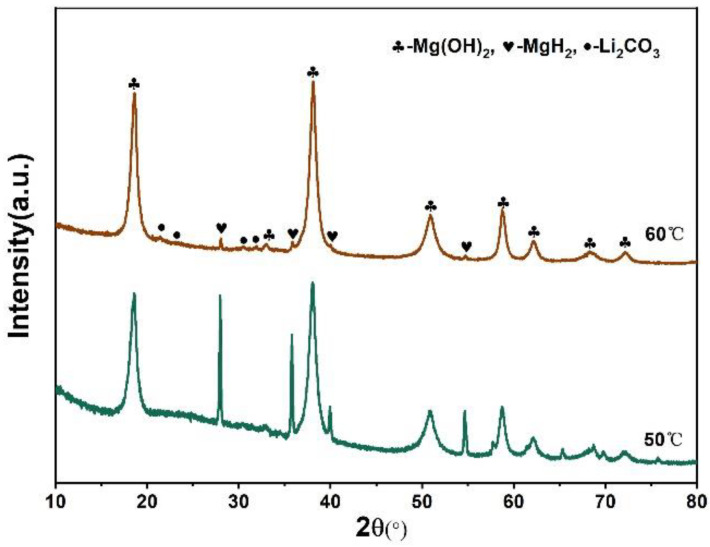
XRD patterns of the hydrolysis byproducts of MgH_2_-3 wt % LiH-BM in deionized water at different bath temperatures.

Furthermore, in order to study the hydrolysis properties of the sample in deionized water, the MgH_2_-3 wt % LiH-BM in deionized water at the different water bath temperatures were investigated as shown in Figure 8. The hydrogen generation yield and hydrogen generation rate are enhanced with the increase of water bath temperatures. One possible reasons is that the higher temperature is conducive to the dissolution of Mg(OH)_2_. Figure 9 shows XRD patterns of the byproducts after hydrolysis in deionized water at water bath temperatures with 50 °C and 60 °C. From Figure 9, the byproducts are composed of MgH_2_, Mg(OH)_2_, and Li_2_CO_3_. In the byproducts, Li_2_CO_3_ may come from CO_2_ absorbed by the LiOH byproduct during the drying and testing process. LiOH is dissolved in water, so there is no associated diffraction peak in XRD [26]. According to the XRD results, the reaction of Mg, MgH_2_ and LiH could be described as follow:

(3) $$\mathrm{LiH}+2H_{2}O\to \mathrm{LiOH}+H_{2}$$

(4) $$\mathrm{LiOH}+{\mathrm{CO}}_{2}\to {\mathrm{LiCO}}_{3}+H_{2}O$$

As the water bath temperature increases from 50 °C to 60 °C, the diffraction peaks intensity of Mg(OH)_2_ increases while the intensity of MgH_2_ decreases. The hydrolysis properties of the sample is obviously dependent on temperature [30]. When the water bath temperature is 60 °C, the final hydrolysis yield reaches about 1510 mL/g until 250 min, with the conversion rate up to about 80%. These results also prove that the suitable water temperature is beneficial to the hydrolysis reaction.

**Figure 10:**
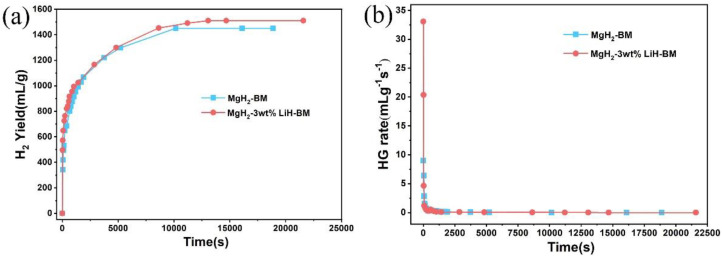
(**a**) Hydrogen generation curves and (**b**) hydrogen generation rate of the MgH_2_ and MgH_2_-3 wt % LiH-BM in water at 60 °C.

We carefully compared hydrogen generation of the MgH_2_-BM and MgH_2_-3 wt % LiH-BM at 60 °C in Figure 10a. As can be seen, the hydrogen generation rate of MgH_2_-3 wt % LiH-BM is improved. The presence of LiH can increase the hydrolysis rate compared to pure MgH_2_. The result can also be seen visually from the hydrogen generation rate diagram, as shown in Figure 10b.

The possible hydrolysis mechanism of the MgH_2_-LiH system in MgCl_2_ aqueous solution was detailed described in previous work of our research group [21]. In pure water, the MgH_2_ hydrolysis reaction leads to an increase −OH concentration on the particles surface. The precipitation of −OH and Mg^2+^ mainly occurs on the surface of particles, so the byproduct Mg(OH)_2_ rapidly deposits on the surface of particles, forming a dense passivation layer. The passivation layer prevents further hydrolysis of MgH_2_. In MgCl_2_ aqueous solution, due to the presence of a large amount of Mg^2+^ in the whole solution system, the Mg^2+^ in solution competes with the MgH_2_ on the surface during the formation of Mg(OH)_2_ precipitation, that is to say, Mg^2+^ in solution combine with OH^−^ on the surface of MgH_2_. In this case, the resulting precipitate is dispersed in the solution rather than forming a passivated layer on the surface.

For hydrolysis properties of the MgH_2_-3 wt % LiH-BM and MgH_2_-3 wt % LiH-G, the MgH_2_-3 wt % LiH-BM has better hydrolysis kinetics than MgH_2_-3 wt % LiH-G. This result is that the reduction of particle size of the samples after planetary ball milling cause the larger specific surface area of the samples, which is more conducive to the rapid hydrolysis reaction [23,30].

For the role of LiH, by XRD, SEM, and reaction byproduct analysis, LiH is uniformly attached to the surface of MgH_2_. LiH hydrolyzes rapidly in water, and the reaction equation is shown as (3): LiH + H_2_O → LiOH + H_2_. Then, Mg^2+^ preferentially binds with OH^−^ ions to form Mg(OH)_2_ precipitation, which is dispersed in water rather than coated on the surface of MgH_2_ nanoparticles. The result is in agreement with reported results for MgLi alloy and MgH_2_-LiNH_2_ composites [8,26].

In addition, in the process of planetary ball milling and grinding in agate mortar, LiH will also enter between MgH_2_ nanoparticles to disperse MgH_2_ and conduce to MgH_2_ fully contact with aqueous solution.

## 4. Conclusions

In this work, the hydrolysis properties of the MgH_2_-LiH system have been studied. The 3 wt % LiH is added into MgH_2_, the hydrogen generation yield can increase about 72% compared to the hydrogen generation yield of MgH_2_-G. The MgH_2_-LiH system hydrolysis is relatively complete, almost 100%, also has the fastest hydrolysis rate in the 1M MgCl_2_ aqueous solution. In a short time, the ball milling reduces the particle size of the samples and cause the larger specific surface area of the samples, which is more conducive to the rapid hydrolysis reaction. The higher water solution temperature is helpful to improve the hydrolysis properties. When the water bath temperature is 60 °C, the final hydrolysis yield reaches to about 1510 mL/g until 250 min.

## Data Availability

Not applicable.
